# Giant congenital lymphedema in an infant with suspected milroy disease (hereditary lymphedema type IA): a case report

**DOI:** 10.3389/fsurg.2026.1902100

**Published:** 2026-10-05

**Authors:** Yoyos Dias Ismiarto, Teuku Aditya Kemal, Sudaryanto, Banan Fajar Rusbi, Frank Don Esparansa Runtuboi

**Affiliations:** Department of Orthopaedic and Traumatology, Sub-Division of Pediatric Orthopaedics, Universitas Padjadjaran, Hasan Sadikin Hospital, Bandung, Indonesia

**Keywords:** elephantiasis, hereditary lymphedema, infant, lymphatic aplasia, milroy disease, surgical debulking

## Abstract

Milroy disease is a rare hereditary lymphatic disorder caused mainly by VEGFR3 mutations and typically presents with lower limb edema. We report a severe unilateral case in a 6-month-old boy with massive right-leg elephantiasis that impaired motor development. Examination and lymphoscintigraphy confirmed lymphatic aplasia and an obstructive pathology. Due to progressive enlargement and functional limitation, surgical debulking with excision of fibrofatty tissue and Z-plasty closure was performed, followed by Complex Decongestive Therapy. Postoperatively, the patient demonstrated good wound healing, a preserved range of motion, and improved function. This case highlights the importance of early multidisciplinary management and the role of surgical intervention in suspected extreme infantile Milroy disease.

## 1 Introduction

Primary lymphedema is a rare disorder caused by abnormal development or function of the lymphatic system, which can occur alone or as part of a syndrome and may be present at birth or develop later in life (1). Kinmonth classified primary lymphedema based on the age of onset. First, the congenital type is relatively rare and presents at birth. Second, the precox type is more common and develops during puberty. Lastly, the tarda type occurs after the age of 35 years (2).

Among the congenital types, Milroy disease (Hereditary Lymphedema Type IA) is a primary congenital lymphedema characterized by the abnormal development of the lymphatic system. It is most commonly associated with mutations in the *VEGFR3* gene, which leads to hypoplasia or aplasia of the initial lymphatic capillaries (3). Clinically, it typically presents at birth with painless, non-pitting edema of the lower extremities. However, in rare instances, the condition can progress into a massive, disfiguring state known as elephantiasis congenital (4).

Recognizing genetic lymphatic disorders at an early stage in newborns with pronounced limb swelling is essential for formulating an effective management strategy. Conservative management, which primarily involves compression therapy and manual lymphatic drainage, is widely recognized as the standard of care. Nevertheless, these methods might fall short for patients dealing with severe or extensive lymphedema (5). In such cases, surgical debulking emerges not merely as an alternative, but as a viable and necessary necessity to preserve the infant's potential for normal physical development (6). Failure to intervene early can lead to severe psychological and functional morbidity, as the infantile elephantiasis becomes a permanent barrier to the infant's quality of life (7).

This case report describes a severe unilateral presentation of suspected Milroy disease with progressive limb enlargement and discusses the transition from conservative management to surgical treatment to facilitate long-term functional development of the affected limb.

## 2 Case presentation

### 2.1 History

A 6-month-old male infant was referred to our specialized center for the evaluation of dramatic and progressive enlargement of the right lower extremity. The parents noted swelling immediately at birth, which was described as rapid, disproportionate growth compared to the rest of the infant's body. By the age of six months, the limb had become markedly enlarged, significantly hindering the infant's ability to roll, sit, or move his leg. The family denied any episodes of fever or skin changes that were suggestive of recurrent cellulitis. There was no known family history of similar swelling, although hereditary patterns were later investigated.

### 2.2 Physical examination

Clinical examination revealed marked circumferential non-pitting swelling in the right lower limb, extending from the knee to the toes. The affected limb measured approximately 30 × 25 × 15 cm at its greatest dimension, with a maximum limb circumference of 67 cm. The skin exhibited significant thickening and a firm texture, which was indicative of advanced fibrosis. A positive Stemmer sign was observed on the dorsal surfaces of the toes. Despite the massive superficial edema, the limb was non-tender, and the distal neurovascular status was preserved with a rapid capillary refill time (<2 s).

The substantial tissue burden resulted in severe restriction of lower-limb mobility. Preoperatively, no appreciable range of motion was observed in the knee or ankle joints, including the ankle dorsiflexion. The mechanical bulk of the affected limb severely limits functional movement and interferes with age-appropriate gross motor development in children. At 6 months of age, the patient was unable to sit or stand independently, which is consistent with delayed gross motor development (Figure 1).

**Figure 1:**
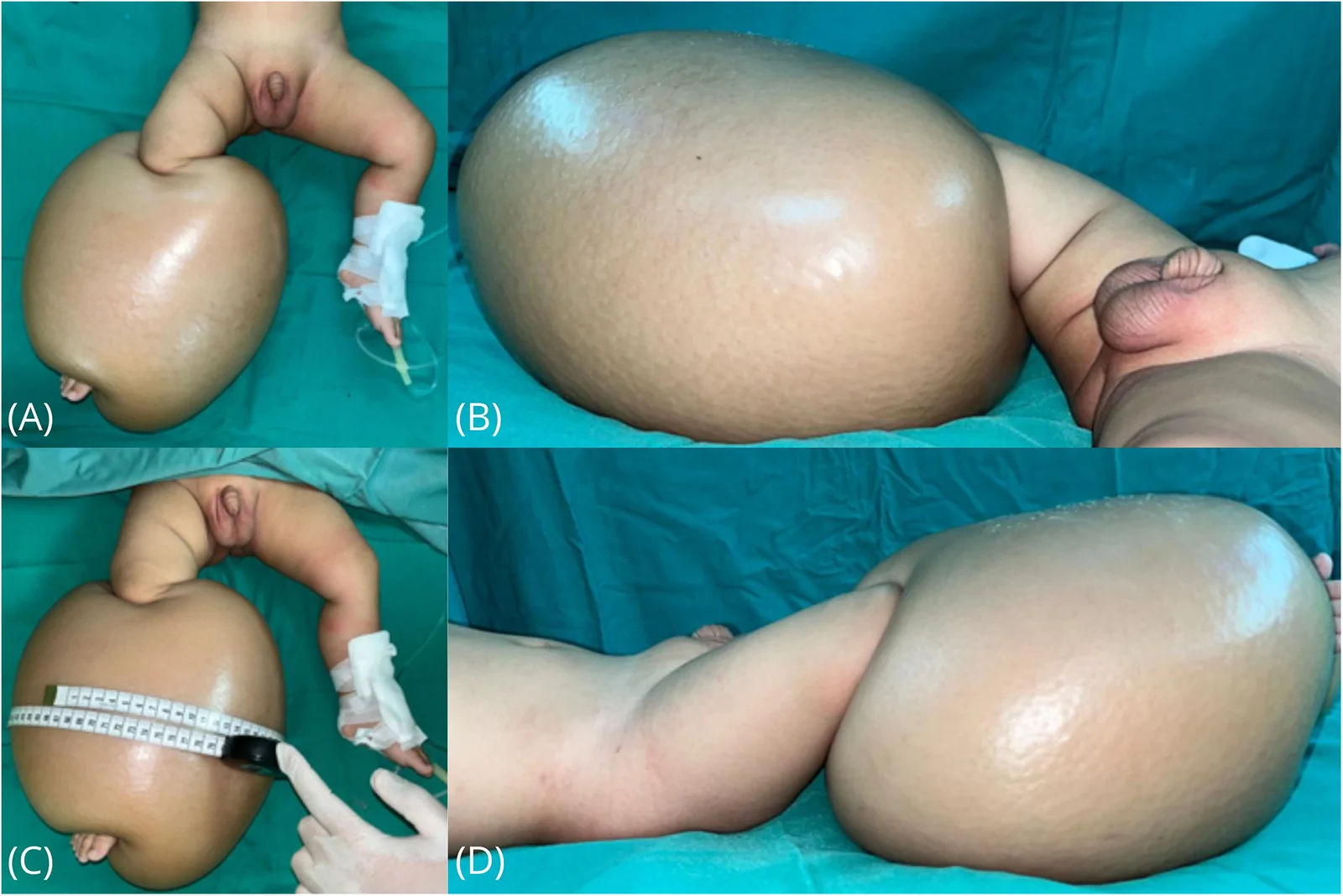
Clinical appearance of the right lower limb demonstrating advanced primary lymphedema. The affected limb exhibited marked circumferential, non-pitting edema extending from the knee to the toes.

### 2.3 Imaging and diagnostic findings

The patient was positioned in the beach chair position at 30 °–45 ° under fluoroscopic guidance. The diagnostic evaluation was strategically designed to quantify the extent of soft tissue hypertrophy and to provide a definitive assessment of lymphatic function. Plain radiography of the right lower extremity revealed marked circumferential soft tissue expansion. While the femur, tibia, and fibula appeared morphologically intact, chronic mechanical stress placed the developing bones at a significant risk for secondary cortical bowing and joint malalignment (Figure 2).

**Figure 2:**
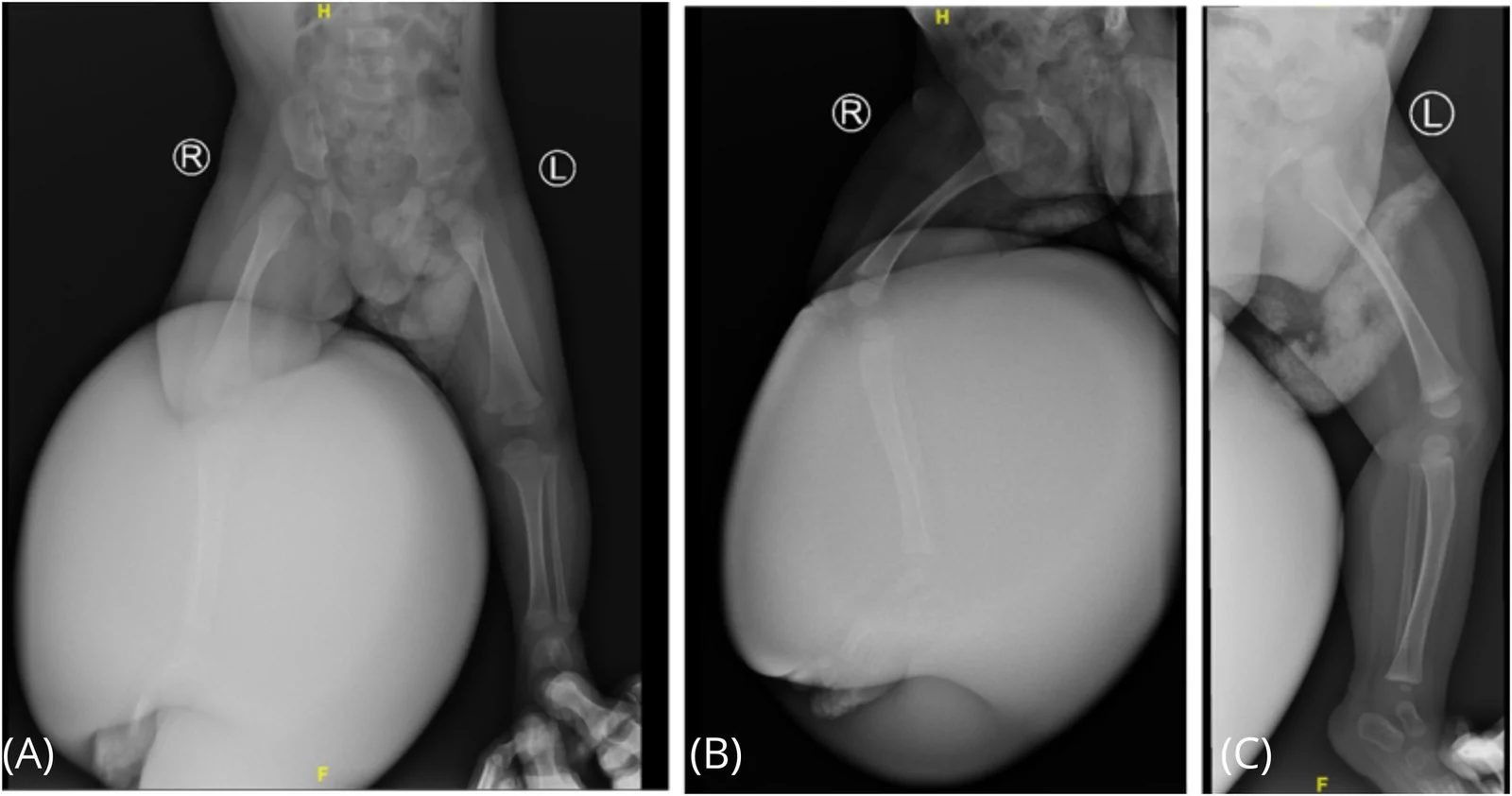
Plain radiographs of the lower extremities in an infant with giant congenital lymphedema secondary to milroy disease (hereditary lymphedema type IA). **(A)** Anteroposterior (AP) view of both lower extremities demonstrating marked massive soft tissue enlargement of the right lower limb; **(B)** Lateral view of the right lower extremity showing extensive circumferential soft tissue swelling extending from the knee to the foot revealed marked circumferential soft tissue expansion; **(C)** Lateral view of the left lower extremity demonstrating relatively preserved soft tissue contour compared to the affected side.

To confirm the clinical suspicion of a primary lymphatic defect, lymphoscintigraphy was performed. The radioisotope tracer demonstrated a classic total obstructive pathology characterized by immediate and significant dermal backflow, a hallmark of valvular incompetence and vessel hypoplasia. Proximal migration of the tracer was near-absent; even at the 60-minute time point, the radioisotope reached the inguinal lymph nodes with markedly diminished intensity. This pattern is consistent with lymphatic hypoplasia seen in Milroy Disease, as opposed to the localized obstruction typically observed in Constriction Band Syndrome (CBS). Taken together, these imaging findings, along with the absence of visible amniotic bands or skin grooves on physical examination, support a clinical diagnosis of suspected Milroy disease with severe congenital lymphedema (Supplementary Figure S1).

### 2.4 Surgical management

Owing to the significant tissue burden and progressive functional impairment, a surgical “debulking-first” approach was chosen to reduce the limb volume and relieve the mechanical stress. The procedure was performed under general anesthesia with continuous hemodynamic monitoring because of the patient's young age and the anticipated risk of substantial fluid and blood loss. Initial decompression was performed before the procedure to reduce the high protein fluid content within the interstitium. Using ultrasound guidance, a small-caliber cannula was inserted into the affected limb to aspirate approximately 280 cc of stagnant lymph fluid. A tumescent solution containing epinephrine was infiltrated into the operative field to minimize the intraoperative bleeding (Supplementary Figure S2).

Longitudinal medial and lateral incisions were made according to the Homan or modified Charles procedure principles, tailored to the infant's anatomy. Extensive hypertrophied fibrofatty tissue was excised from the anterior part of the calf (Supplementary Figure S3). The abnormal deep fascial tissue was partially excised along the medial-to-lateral calf horizontally from the superficial aspect of the crural fascia, while the underlying muscle compartments were preserved. The procedure involved excising only the visibly thickened and abnormal fascia while carefully maintaining the integrity of the underlying muscle tissue (Figure 3).

**Figure 3:**
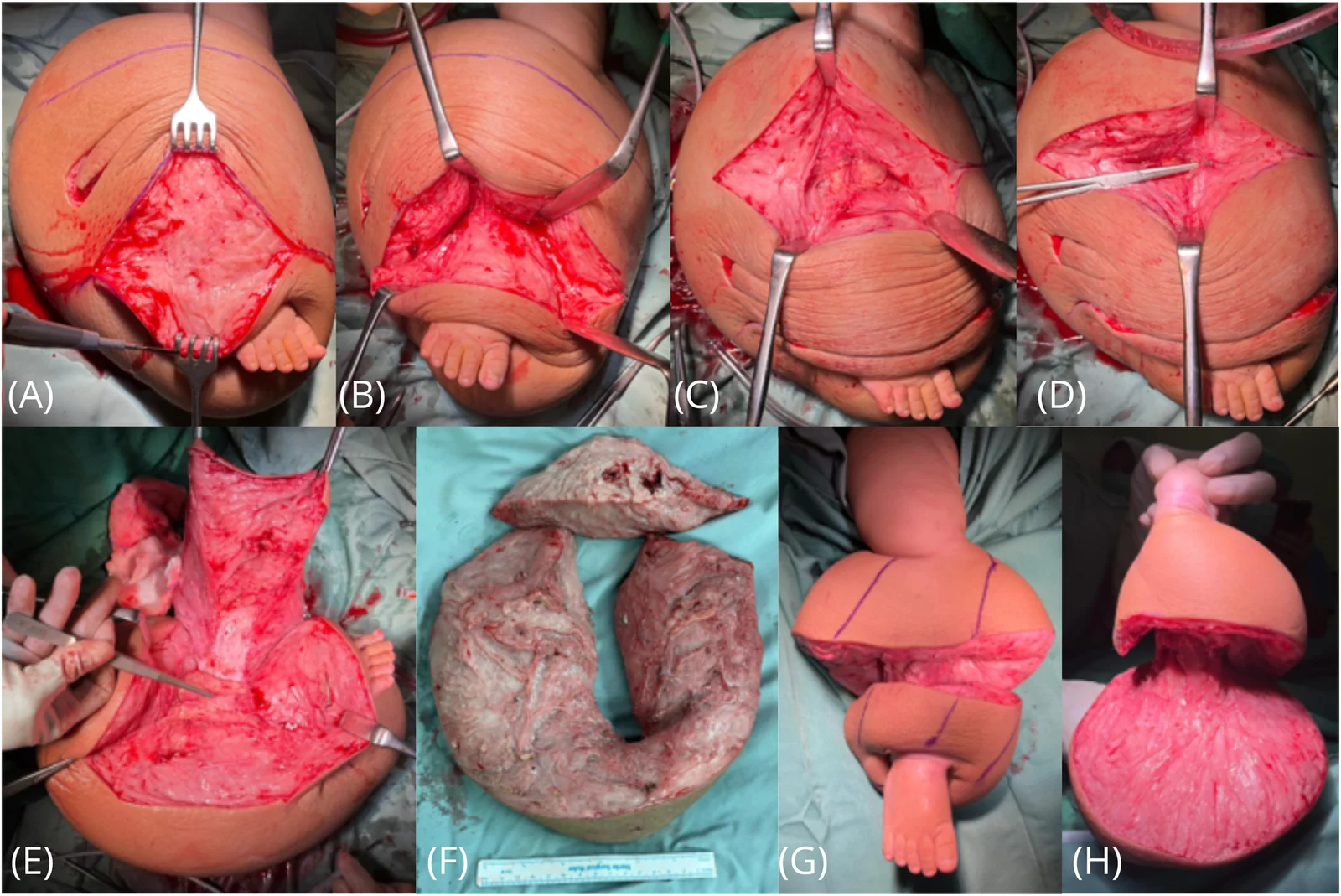
Intraoperative findings during debulking surgery of the right lower limb. **(A–D)** Sequential steps of excision of the hypertrophied fibrofatty tissue. **(E)** Intraoperative appearance of enlarged lymph nodes. **(F)** Resected fibrofatty tissue specimen. **(G,H)** Final appearance of the right lower limb after completion of the debulking procedure.

Major neurovascular structures were identified and preserved during dissection. Hemostasis was achieved using bipolar electrocautery. The total weight of the resected fibrofatty and fascial tissue was 200 g. The estimated intraoperative blood loss was 1,800 cc, and blood transfusion was not required. The total operative time was six hours. Following volume reduction, the remaining skin envelope was assessed for redundancy and tension. Multiple Z-plasties were incorporated into the primary closure at the anterior calf to interrupt linear scar formation, reduce the risk of postoperative contracture, and provide additional skin length across areas expected to undergo growth (Figure 4). No drain was placed at the end of the procedure.

**Figure 4:**
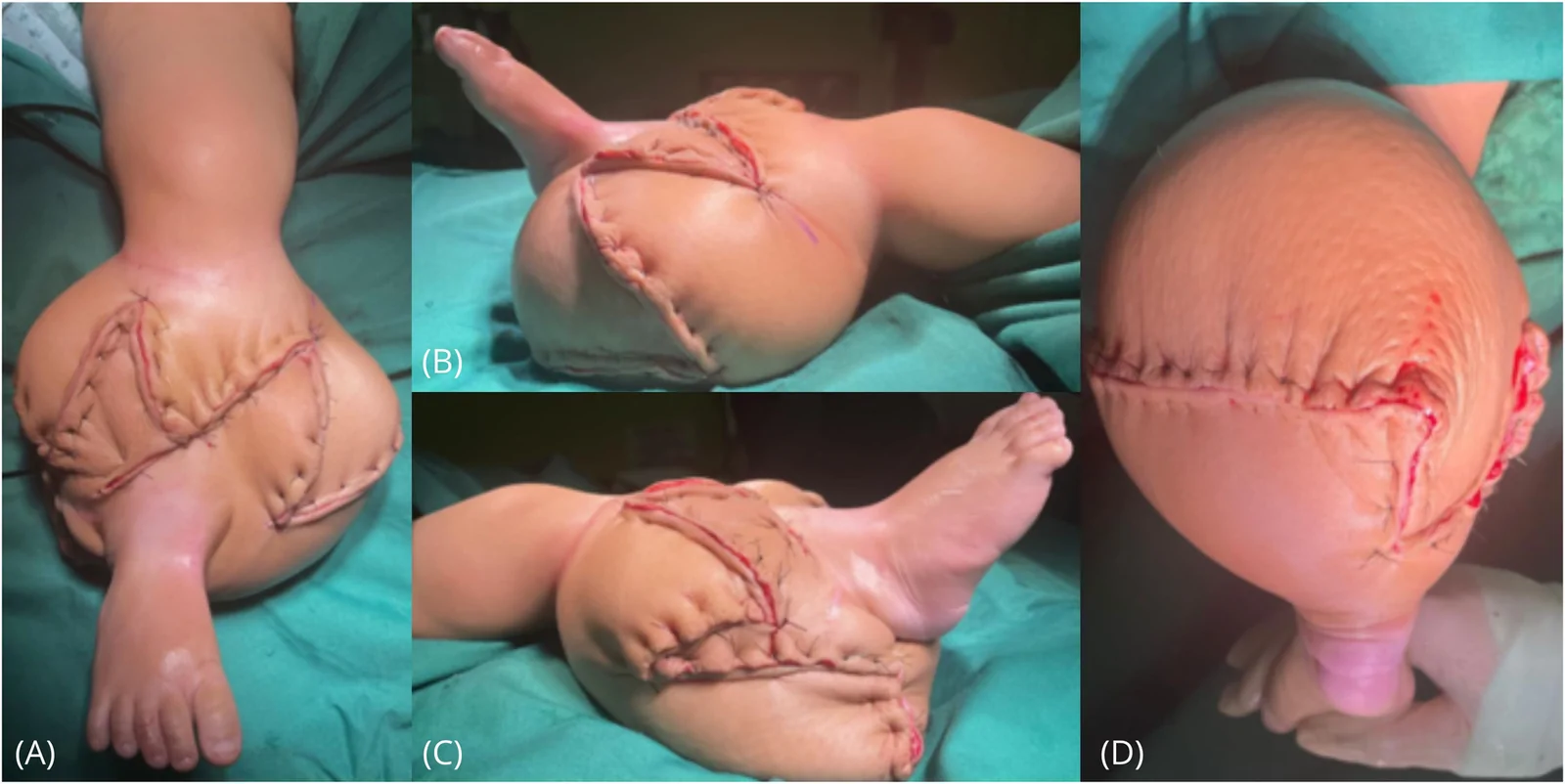
Intraoperative appearance after wound closure with multiple Z-plasties following debulking surgery. **(A)** Top view of the affected limb showing the surgical incisions and sutures. **(B)** Left-side view demonstrating the postoperative sutured wounds and residual swelling. **(C)** Right-side view showing the surgical closure and postoperative edema. **(D)** Bottom view of the sutured surgical site with the foot visible distally.

Postoperatively, the patient was monitored for ten days for hemodynamic stability, wound condition, drainage, and signs of infection or neurovascular compromise. A compression dressing was applied, and physiotherapy was initiated when clinically appropriate. Lymphovenous anastomosis (LVA) was considered a subsequent reconstructive option; however, it was not performed during the initial procedure because the initial priority was to reduce the substantial tissue burden before considering definitive lymphatic reconstruction at a later stage.

### 2.5 Follow up

Postoperative care was centered on strict elevation of the limb and commencement of Complex Decongestive Therapy (CDT) to maintain volume reduction. On the 15th postoperative day, the patient presented for a scheduled evaluation (Supplementary Figure S4). Subjectively, the infant experienced mild surgical site pain and exhibited moisture at the incision site. Clinical examination of the right leg revealed a well-healing surgical wound with minimal discharge and no active bleeding. The distal neurovascular status remained intact, as evidenced by normal distal sensation and a capillary refill time of less than two seconds, although the range of motion at the knee and ankle remained restricted due to postoperative discomfort. Management at this stage involved changing the dressing and removing the sutures. The patient was subsequently referred for coordinated long-term follow-up with the Vascular and Plastic Surgery teams to monitor recurrence and functional limb development.

At the two-month postoperative follow-up, the patient demonstrated favorable early clinical findings following the debulking surgery (Supplementary Figure S5). The surgical scars had matured well and appeared soft and flat, with no evidence of hypertrophic scarring, keloid formation, or significant contracture at this time. Compared with the preoperative examination, the patient demonstrated improved mobility of the knee and ankle at two months postoperatively. No new distal or neurovascular deficits were identified. However, no clear improvement in gross motor development was observed. The reduction in limb bulk was clinically maintained by continued compression therapy and appropriate limb positioning. The postoperative limb dimensions were 20 × 15 × 10 cm compared with the preoperative dimensions of 30 × 25 × 15 cm. No wound complications or clinically significant recurrent swelling were identified during this early follow-up. The patient remained under multidisciplinary follow-up with the Vascular and Plastic Surgery teams, with continued monitoring of limb size, joint range of motion, motor development, and recurrence. Given the two-month follow-up period, these findings represent early postoperative outcomes, and longer-term surveillance is required to assess recurrence, growth-related changes, contractures, and functional development.

## 3 Discussion

This case represents a severe form of congenital lymphedema that presented with progressive unilateral lower limb enlargement during infancy. Lymphoscintigraphy revealed impaired lymphatic drainage, prominent dermal backflow, and reduced visualization of lymphatic vessels, consistent with lymphatic dysfunction and hypoplasia. The early onset, predominantly lower-extremity involvement, and absence of clinical or imaging features suggestive of vascular malformation or constriction band syndrome supported the clinical suspicion of Milroy disease. However, in the absence of *FLT4/VEGFR3* genetic testing, the diagnosis should be regarded as clinically suspected rather than genetically confirmed. Milroy disease is associated with abnormalities in the VEGFR3 signaling pathway, which plays an important role in lymphatic vessel development and maturation (3). Inadequate development of lymphatic vessels leads to impaired lymphatic drainage and accumulation of protein-rich interstitial fluid in the affected tissues. Persistent lymph accumulation can stimulate fibroblasts and other inflammatory cells, resulting in progressive tissue remodeling characterized by fibrosis, adipose tissue deposition, and skin thickening (8, 9). These pathological changes may contribute to progressive tissue enlargement and increased limb volume over time, as observed in the present case.

Nevertheless, given the marked unilateral limb enlargement observed during infancy, several congenital vascular and overgrowth disorders should be considered in the differential diagnoses. Venolymphatic malformation was considered because it can present with limb swelling due to the abnormal development of venous and lymphatic vessels. These lesions may be associated with pain, bleeding, infection, or other vascular abnormalities (10). However, in this case, there was no pain, infection, or imaging evidence of a characteristic venous or combined venolymphatic malformation, making this diagnosis less likely.

The PIK3CA-related overgrowth spectrum (PROS) was also considered because it may present with asymmetric overgrowth and can involve vascular, lymphatic, skeletal, and adipose tissues (11). Unlike the predominantly lymphatic phenotype observed in this case, PROS typically presents with segmental or disproportionate overgrowth involving multiple tissue types and may be accompanied by associated vascular or other congenital anomalies (12). The absence of characteristic segmental overgrowth or associated anomalies in this patient made PROS less likely.

Klippel–Trénaunay syndrome (KTS) is another important differential diagnosis because it commonly presents with unilateral limb overgrowth. KTS is characterized by a combination of capillary malformations, typically port-wine staining, venous abnormalities such as varicosities or venous malformations, and hypertrophy of soft tissue and bone (13). In the present case, the absence of characteristic capillary and venous abnormalities made the diagnosis of KTS less likely.

The mainstay of management for primary lymphedema generally involves complete decongestive therapy (CDT), including manual lymphatic drainage, compression therapy, exercise as appropriate, and skin care (14). CDT aims to reduce limb volume, maintain skin integrity, and prevent complications. In the present case, surgical debulking was performed initially because of the severe and progressive enlargement of the affected limb (15). Following surgery, CDT was initiated as part of postoperative management to support limb-volume control, maintain the surgical outcome, and facilitate functional recovery. However, in patients with severe or progressive lymphedema, particularly when substantial tissue enlargement has developed, conservative treatment may not provide sufficient reduction in limb size or functional improvement (16).

In the present case, the decision to proceed with surgical debulking was based on the severity and progressive enlargement of the affected limb, with the aim of improving mobility and facilitating age-appropriate motor development in the child. Severe limb enlargement may interfere with normal movements and potentially contribute to musculoskeletal complications. In a study of 38 patients with lower-extremity lymphedema, 30 experienced significant and sustained reductions in limb size, accompanied by improved function and fewer episodes of cellulitis (17). Similarly, the present case demonstrated a reduction in limb size and improved range of motion following surgical intervention.

In addition to debulking procedures, physiological surgical approaches, such as lymphaticovenous bypass or anastomosis (LVB/LVA) and vascularized lymph node transfer (VLNT), have been described for the treatment of lymphedema (16). Evidence supporting microsurgical lymphatic reconstruction in primary lymphedema, particularly in pediatric patients, remains limited and is largely based on observational studies. Nevertheless, available reports suggest that these procedures may reduce limb volume, decrease episodes of cellulitis, and improve quality of life in selected patients (18).

Excisional debulking primarily addresses the solid component and excess tissue of advanced lymphedema, whereas physiological procedures aim to improve the lymphatic drainage. Therefore, these approaches may be used in combination or in a staged manner to address both components of the disease (19). In the present case, debulking was performed as the initial procedure to achieve a substantial reduction in limb volume. A second-stage LVA is planned to further improve lymphatic drainage and complement the volume-reducing effect of the initial debulking procedure.

Early consideration of surgical intervention may be important in selected patients with severe and progressive limb enlargement, particularly when the condition interferes with mobility and motor development in children. A multidisciplinary approach involving relevant surgical and rehabilitation specialists may facilitate adequate volume reduction while preserving neurovascular structures and optimizing postoperative function (20). Although surgical treatment does not correct the underlying lymphatic developmental abnormality, reduction of excessive limb volume may improve mobility and facilitate age-appropriate motor development, including crawling and standing.

This case report has several limitations. First, genetic testing for FLT4/VEGFR3 was not performed; therefore, the diagnosis of Milroy disease remains clinically suspected rather than genetically confirmed. Second, although surgical debulking resulted in improvements in limb size and range of motion, planned second-stage lymphaticovenous anastomosis (LVA) has not yet been performed. Consequently, its potential contribution to long-term lymphatic drainage and limb volume control cannot yet be assessed. Third, as this is a single case, the long-term durability of the clinical and functional outcomes and generalizability of this approach to other patients with severe congenital lymphedema remain uncertain.

In conclusion, Milroy disease presenting with severe congenital elephantiasis requires early diagnostic evaluation, including lymphoscintigraphy, when appropriate. A multidisciplinary approach is essential for optimizing functional outcomes and addressing the clinical needs of affected patients and their families. Genetic counseling is an important component of management. While conservative therapy remains the mainstay of treatment for typical lymphedema, surgical debulking may be considered in selected patients with severe or massive limb enlargement when conservative measures are insufficient. In such cases, surgical treatment may help reduce limb volume, improve function, and facilitate physical growth.

## Data Availability

The original contributions presented in the study are included in the article/Supplementary Material, further inquiries can be directed to the corresponding author.
