# Supplementary material for: Giant congenital lymphedema in an infant with suspected milroy disease (hereditary lymphedema type IA): a case report
Source: Front Surg. 2026 Oct 5;13:1902100. doi: 10.3389/fsurg.2026.1902100 (PMC13718031; doi:10.3389/fsurg.2026.1902100)
Supplement: Supplementary file 1 [file Table1.docx]

Supplementary Material

# 1 Supplementary Figures

**Supplementary Figure 1.** The imaging features, together with the absence of visible amniotic bands or circumferential skin grooves on physical examination, favored the diagnosis of Milroy disease complicated by secondary giant elephantiasis congenital.

**Supplementary Figure 2.** Intraoperative preparation prior to debulking surgery, ultrasound-guided aspiration of stagnant lymphatic fluid using a small-caliber cannula.

**Supplementary Figure 3.** Intraoperative marking and execution of longitudinal medial and lateral incisions during debulking surgery.

**Supplementary Figure 4.** Clinical appearance of the right lower limb on postoperative day 15.

**Supplementary Figure 5.** Clinical appearance of the right lower limb on postoperative 2 months.
